# Progesterone at high doses reduces the growth of U87 and A172 glioblastoma cells: Proteomic changes regarding metabolism and immunity

**DOI:** 10.1002/cam4.3223

**Published:** 2020-06-26

**Authors:** Meric A. Altinoz, Yasemin Ucal, Muazzez C. Yilmaz, İrem Kiris, Ozan Ozisik, Ugur Sezerman, Aysel Ozpinar, İlhan Elmaci

**Affiliations:** ^1^ Department of Medical Biochemistry School of Medicine Acibadem Mehmet Ali Aydinlar University Istanbul Turkey; ^2^ Medical Genetics Aix Marseille University, Inserm, MMG Marseille France; ^3^ Department of Biostatistics and Medical Informatics Acibadem Mehmet Ali Aydinlar University Istanbul Turkey; ^4^ Department of Neurosurgery Acibadem Maslak Hospital and School of Medicine, Acibadem Mehmet Ali Aydinlar University Istanbul Turkey

**Keywords:** cell line, glioblastoma, neuroendocrine treatment, progesterone, proteomic

## Abstract

While pregnancy may accelerate glioblastoma multiforme (GBM) growth, parity and progesterone (P4) containing treatments (ie, hormone replacement therapy) reduce the risk of GBM development. In parallel, low and high doses of P4 exert stimulating and inhibitory actions on GBM growth, respectively. The mechanisms behind the high‐dose P4‐suppression of GBM growth is unknown. In the present study, we assessed the changes in growth and proteomic profiles when high‐dose P4 (100 and 300 µM) was administered in human U87 and A172 GBM cell lines. The xCELLigence system was used to examine cell growth when different concentrations of P4 (20, 50, 100, and 300 µM) was administered. The protein profiles were determined by two‐dimensional gel electrophoresis in both cell lines when 100 and 300 µM P4 were administered. Finally, the pathways enriched by the differentially expressed proteins were assessed using bioinformatic tools. Increasing doses of P4 blocked the growth of both GBM cells. We identified 26 and 51 differentially expressed proteins (fc > 2) in A172 and U87 cell lines treated with P4, respectively. Only the pro‐tumorigenic mitochondrial ornithine aminotransferase and anti‐apoptotic mitochondrial 60 kDa heat shock protein were downregulated in A172 cell line and U87 cell line when treated with P4, respectively. Detoxification of reactive oxygen species, cellular response to stress, glucose metabolism, and immunity‐related proteins were altered in P4‐treated GBM cell lines. The paradox on the effect of low and high doses of P4 on GBM growth is gaining attention. The mechanism related to the high dose of P4 on GBM growth can be explained by the alterations in detoxification mechanisms, stress, and immune response and glucose metabolism. P4 suppresses GBM growth and as it is nontoxic in comparison to classical chemotherapeutics, it can be used as a new strategy in GBM treatment in the future.

## Graphical abstract

GBM cell line impedance alterations when different concentrations of Progesterone were administered.

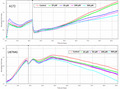

## 1 INTRODUCTION

Glioblastoma multiforme (GBM) has an extremely grave prognosis with approximately 15 months of overall survival despite the golden standard of care.^1^, ^2^ Therefore, the proposal for innovative treatment regimes is of crucial importance. Despite some controversies, many available studies demonstrate that female gender, parity, and hormone replacement treatment reduce the risk of GBMs, while the reverse is true for meningiomas.^1^, ^2^ In pregnancy, the levels of progesterone (P4) gradually increase up to 200‐fold to around 160 µM. Even though pregnancy may accelerate the growth of gliomas, the most angiogenic brain tumors are encountered lesser during the last trimester when the natural 17‐OH‐progesterone levels reach peak levels.^2^, ^3^ This paradox can be explained by the fact that low‐dose P4 stimulates the growth of gliomas, while sustained exposure to high‐dose P4 may reduce glioma cell growth.^2^, ^4^, ^5^ Although P4 is a steroid hormone, it has peculiar immune‐stimulating activities for protecting both the mother and the fetus from potential infections. Additionally, medroxyprogesterone acetate (MPA)—a 17‐OH‐progesterone analog—has immunostimulant activities in cancer.^2^ The MPA treatment can block tumor growth, enhance procarbazine chemosensitivity, and induce cell kill and prevent invasion in a C6 rat model, U87 and U251 human GBM cells, respectively.^1^, ^6^, ^7^ Besides, it was shown that MPA and P4 modified the vascularization pattern and reduced tumor volumes in intracranially implanted glioma models.^1^, ^5^, ^6^, ^8^, ^9^ In electron microscopical studies, MPA induced autophagy and myelinosis, suggestive of lysosomal phospholipid accumulation in C6 glioblastoma cells.^7^ Some groups proposed that P4‐mediated inhibition of PI3‐kinase cascade contributes to its antiproliferative effects in GBM monolayer cell cultures.^5^ However, low doses of PI3‐kinase inhibitors attenuated anti‐invasive actions of MPA in human glioma three‐dimensional spheroid cultures.^1^ To understand the effects in a clear pattern, we selected to study the effects of the native P4 on the proteomic profiles of two different GBM cells at two different doses (100 and 300 µM), which are close to nontoxic concentrations of P4 in the placenta and were previously shown to be efficient in stimulating cell kill in human GBM cells.^1^, ^2^

## 2 MATERIALS AND METHODS

### 2.1 Chemicals and cell culture experiments

All the chemicals and reagents were obtained from Sigma Aldrich (St. Louis, USA) if not stated otherwise. Human glioblastoma cell lines U87 (HTB‐14) and A172 (CRL‐1620) were purchased from the American Type Culture Collection (ATCC) (Manassas, VA, USA). Twenty mM P4 (Sigma‐P8783) was prepared as stock solutions dissolved in ethanol and sterile medium. Before the cell culture studies, the stock solution was diluted to the desired P4 concentrations (20, 50, 100, and 300 µM). As a basic medium, Gibco DMEM (Dulbecco's Modified Eagle's Medium) GlutaMax (500 mL) was employed and 1% (5 mL) penicillin‐streptomycin (10 000 U/mL) was added to this medium to prevent bacterial contamination. The medium was divided into volumes of 45 mL. Fetal bovine serum (FBS) 10% (5 mL) was added to prepare a complete medium (45 mL). The incubator, in which the flasks were kept, had a humidified atmosphere of 5% CO_2_ at 37°C. After the cells covered 70%‐80% of the plate surface, cells were harvested with trypsin‐EDTA and passaged. Trypan blue (Thermo Fisher Scientific, USA) exclusion and a Thoma chamber (Isolab Laborgeräte GmbH, Germany) were used for vital cell identification and counting, respectively. The vital cells (1 × 10^6^) were passaged into plates containing 8 mL of complete medium.

### 2.2 Cell proliferation assessment

CELLigence Real‐Time Cell Analyzer (RTCA) dual‐purpose (DP) device (ACEA) is based on electrical impedance to assess the changes in cell proliferation, morphology, and binding quality in real‐time without labeling. For background measurement, 100 µL medium was added for each well and incubated for 15 minutes. Glioblastoma cell lines—U87 and A172—were seeded in complete medium in 96‐well E‐plate at a density of 5000 cells/well and 7500 cells/well, respectively. Both cell lines were allowed to adhere to the surface for 24 hours in a humidified 37°C incubator with 5% CO_2_. The logarithmic proliferation of the cells was observed using the RTCA device. In the middle of the logarithmic growth phases of U87 and A172 glioma cells, the complete medium was carefully removed. Four different concentrations of P4 and an equivalent volume of 2% ethanol (in U87 control and A172 control cells) were added in no‐FBS‐containing medium at the end of the 18th and 22nd hours, respectively.

The E‐Plates were then inserted into the xCELLigence device to monitor real‐time cell growth. After 72 hours of P4 administration, the experiment was finalized and data were analyzed with RTCA 2.1.0 software. After the 72nd hour, the cells were collected by a scraper, and the pellet was kept in − 80°C for proteomics analysis. All the cell culture experiments were designed as triplicates.

### 2.3 Sample preparation for proteomic analyses

The frozen pellets were lysed with protein extraction buffer (8 mol/L urea, 4% (w/v) CHAPS, 40 mmol/L Tris, 0.2% (w/v) Bio‐Lyte 4/7 Ampholite, 1 mM DTT). Methanol/chloroform precipitation was employed to eliminate salts and other contaminants from the protein sample. The rehydration buffer (7 M urea, 2 M thiourea, 150 U/mL Benzonase, 4% CHAPS, 0.1% Bromophenol blue) was used to dissolve the precipitated protein pellet. Dissolved samples were centrifuged (10 000 g, 30 minutes) and 2D Quant kit (GE Healthcare Life Sciences) was used for protein concentration assessment.

### 2.4 Two‐dimensional gel electrophoresis experiments

The cell lysate (100 μg of proteins) with a fluorescent dye (HPE lightning red dye, Serva Electrophoresis GmbH, Heidelberg, Germany) was administered to 17 cm nonlinear IPG strips pH 3‐10 (Serva Electrophoresis GmbH, Heidelberg, Germany), followed by overnight rehydration at room temperature. The isoelectric focusing (IEF) condition of the strips was as follows: 100 V for 2 hours, 200 V for 1 hours, 500 V for 1 hours, 1000 V for 1 hours, 2000 V for 2 hours, and 5000 V for the time required to obtain 45 000 Vh in total. Equilibration buffer (6 M urea, 30% glycerol, 2% SDS, 50 mM Tris (pH 8.8), and 2% dithiothreitol (DTT)) was applied for 15 minutes to the focused strips. Then the same equilibration buffer including 2.5% iodoacetamide rather than DTT was applied for 15 minutes. The separation in the second dimension was applied on 12.5% SDS‐PAGE on a horizontal electrophoresis unit (HPE Blue Horizon, Serva Electrophoresis GmbH, Heidelberg, Germany) at 15°C using the following voltage parameters: 100 V for 30 minutes, 200 V for 30 minutes, 300 V for 10 minutes, 1000 V for 4 hours, and 1500 V for 40 minutes. The scanning was performed by a Typhoon FLA 9500 (GE Healthcare Life Sciences) at a resolution of 100 μm. Next, the gels were stained overnight in Colloidal Coomassie Blue (CBB) solution (500 mL CBB/gel).^10^ The gel images were processed by PDQuest Advanced 8.0.1 Software (Biorad Laboratories, Hercules, CA). Three gel images from each cell line (U87 and A172) with 100 µM P4, 300 µM P4 and without P4 were uploaded to the software. The master gel was selected by the software and detected spots in all the gels were automatically matched to the master gel. The differences in intensities between corresponding spots on each set of gels were determined. Differential spot intensities (>2‐fold change (fc)) were further assessed by the Student's t test (*P* < .05 was considered as significant).

### 2.5 Protein identification by mass spectrometry

Spots with differential intensities were cut from the gel and destained in a wash buffer (50% acetonitrile, 50% 50 mM ammonium bicarbonate buffer). After destaining, spots were washed with water and dehydrated (100% acetonitrile). Then the spots were reduced and alkylated with 10 mM DTT and 55 mM iodoacetamide, respectively. The spots were then dehydrated with 100% acetonitrile and dried. The spots were incubated overnight at 37°C with 125 ng of trypsin (Promega, Madison, Wisconsin, USA). The peptides were prepared for MALDI‐TOF/TOF MS by Stage tips.^11^ MALDI‐TOF/TOF MS analyses were performed using RapiFLEX MALDI Tissuetyper™ (Bruker Daltonics, Bremen, Germany) instrument. The MALDI matrix was prepared according to Ucal and Ozpinar (2018).^12^ Peptide mixture (0.8 μL) was deposited on an AnchorChip plate (Bruker Daltonics, Bremen, ’Germany), allowed to air‐dry and covered with 0.5 μL MALDI matrix. The mass spectra were obtained within the mass range of 700‐2600 Da and calibrated using the peptide standards (Peptide Std II, Bruker Daltonics, Bremen, Germany). The flexAnalysis 3.3 program (Bruker Daltonics, Bremen, Germany) was used for creating peaks lists. The peak lists were searched against the Uniprot human reference proteome database using MASCOT (Matrix Science). In the database, taxonomy: Homo sapiens (human); fixed modification: carbamidomethyl (C); variable modification: methionine, monoisotopic; peptide mass tolerance: 0.2 Da; peptide charge state: +1; maximum missed cleavages: 1 were selected as parameters.

### 2.6 Pathway enrichment analysis

For the analysis of pathways enriched by the differing proteins in two different GBM cell lines after two different doses, we used pathfindR R package (version 1.3.0).^13^ Next, we ran pathfindR with default parameters except that searching for Reactome pathways ^14^ that contained at least three significant genes.

## 3 RESULTS

### 3.1 Cell proliferation determined by xCelligence system

**FIGURE 1:**
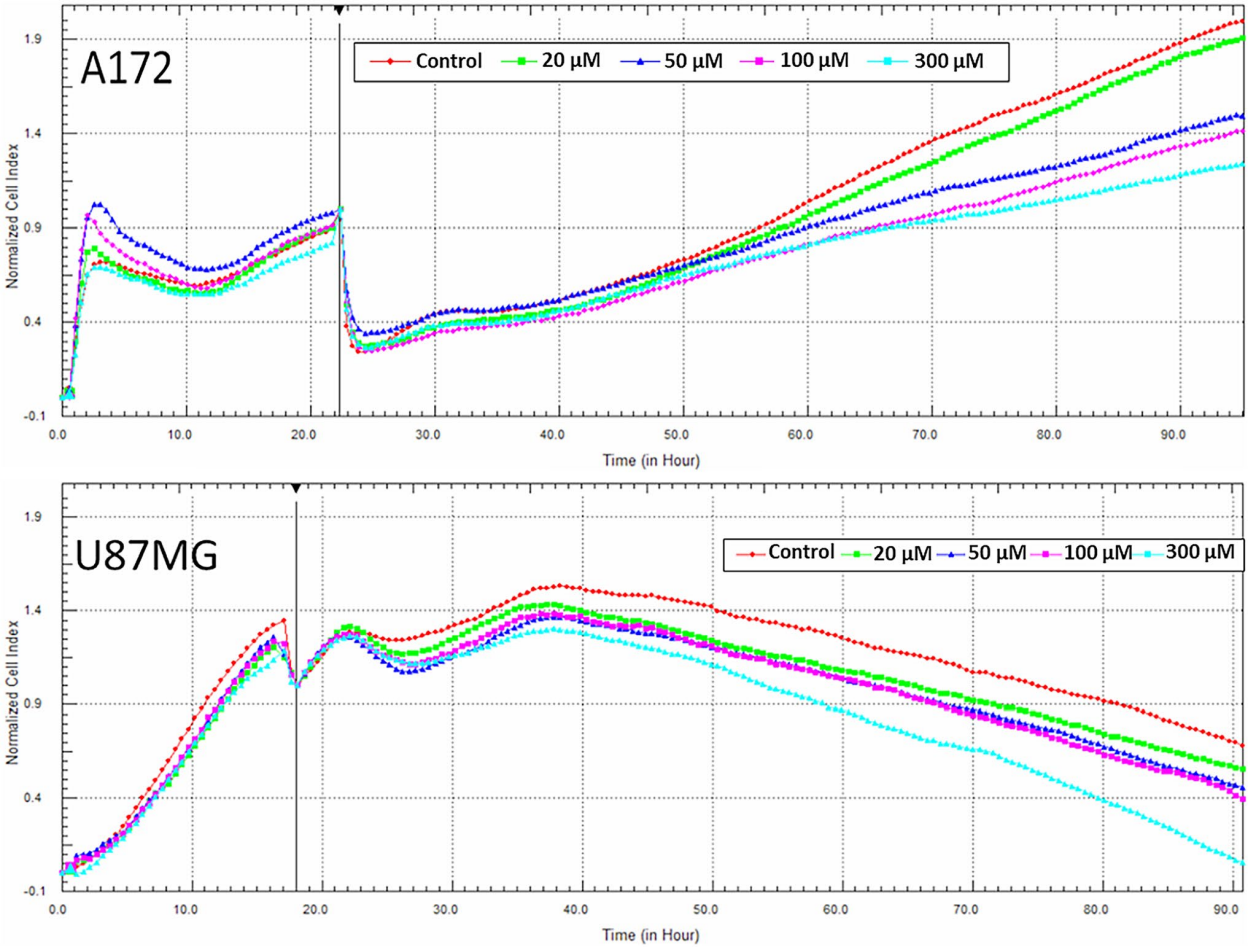
Monitoring of GBM cell line impedance alterations using the xCELLigence system. A, A172 cells were monitored in 96‐well E‐plate at the density of 7500 cells/well. Four different concentrations of P4 (20, 50, 100, and 200 µM) and an equivalent volume of 2% ethanol (control) were added in cell medium at the end of 22nd hour. B, U87 cells were monitored in 96‐well E‐plate at the density of 5000 cells/well. Four different concentrations of P4 (20, 50, 100, and 200 µM) and an equivalent volume of 2% ethanol (control) were added in cell medium at the end of 18th hour

The xCelligence system was employed to investigate impedance alterations by the cytotoxic effect of P4 on U87 and A172 GBM cell lines. Twenty µM, 50, 100, and 300 µM concentrations of P4 were administered for 72 hours to both cell lines (Figure 1). In our experiments, A172 cells continued to grow until 72nd hour of culturing, while U87 cell growth declined around after 40th hour of culture. P4 reduced the growth of A172 cells after about 60th hour of culture and accelerated the growth reduction in U87 cells after about 40th hour of culture. All the tested doses inhibited growth and the degree of inhibition correlated with the applied dosage. The most cytotoxic doses in both cell lines—100 µM and 300 µM—were chosen for further proteomic analysis.

### 3.2 Analysis of differential protein expression in U87 and A172 human GBM cell lines

**FIGURE 2:**
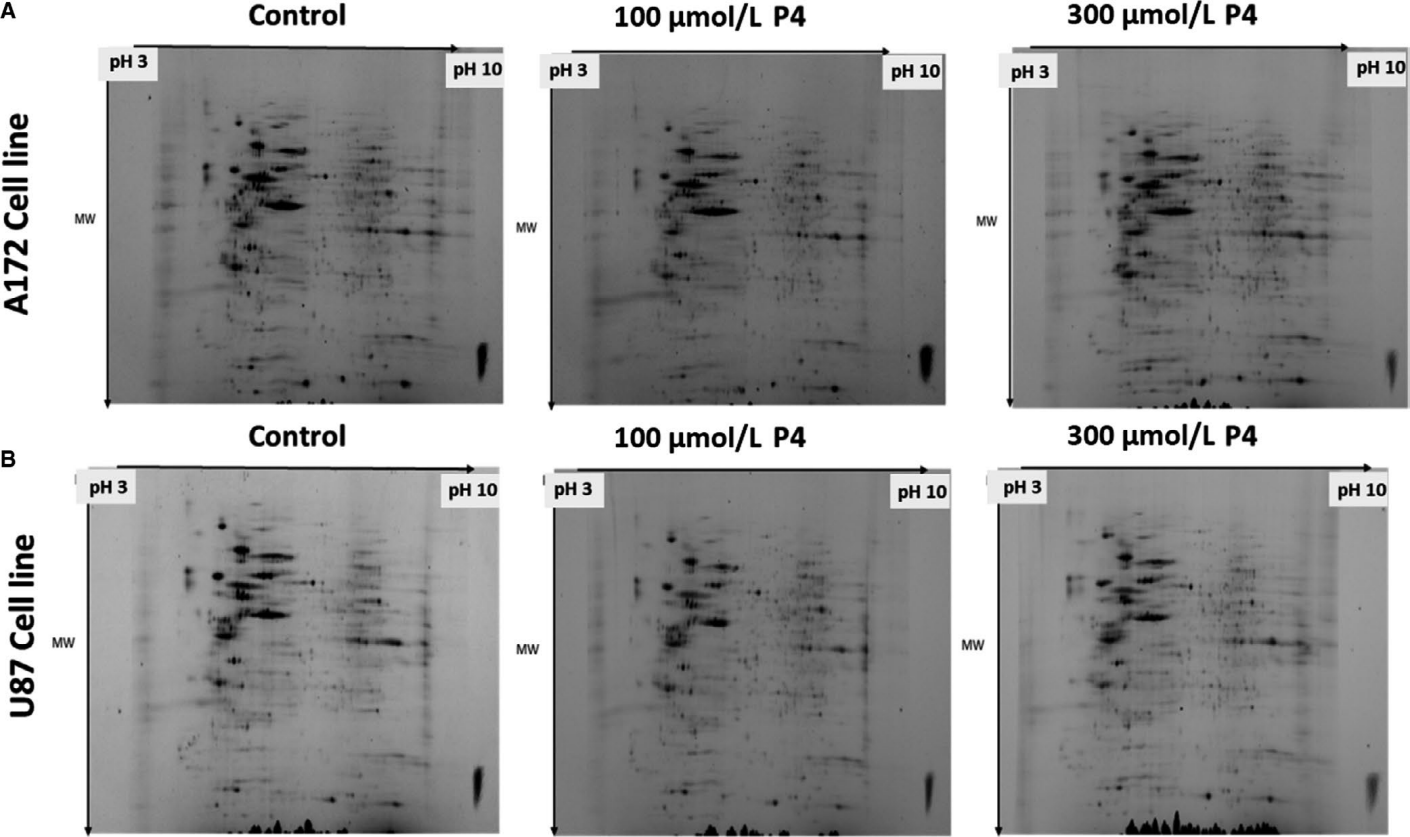
Representative gel images including protein spots from (A) 172 cell line (control, 100 µM P4, 300 µM P4) (B) U87 cell line (control, 100 µM P4, 300 µM P4) identified by 2D gel electrophoresis

**FIGURE 3:**
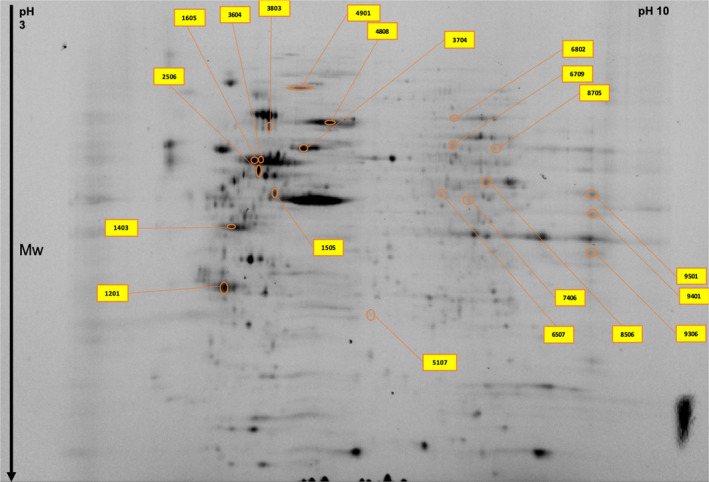
Significantly altered protein spots in the A172 cell line master gel image obtained by two‐dimensional gel electrophoresis proteomics analysis

**FIGURE 4:**
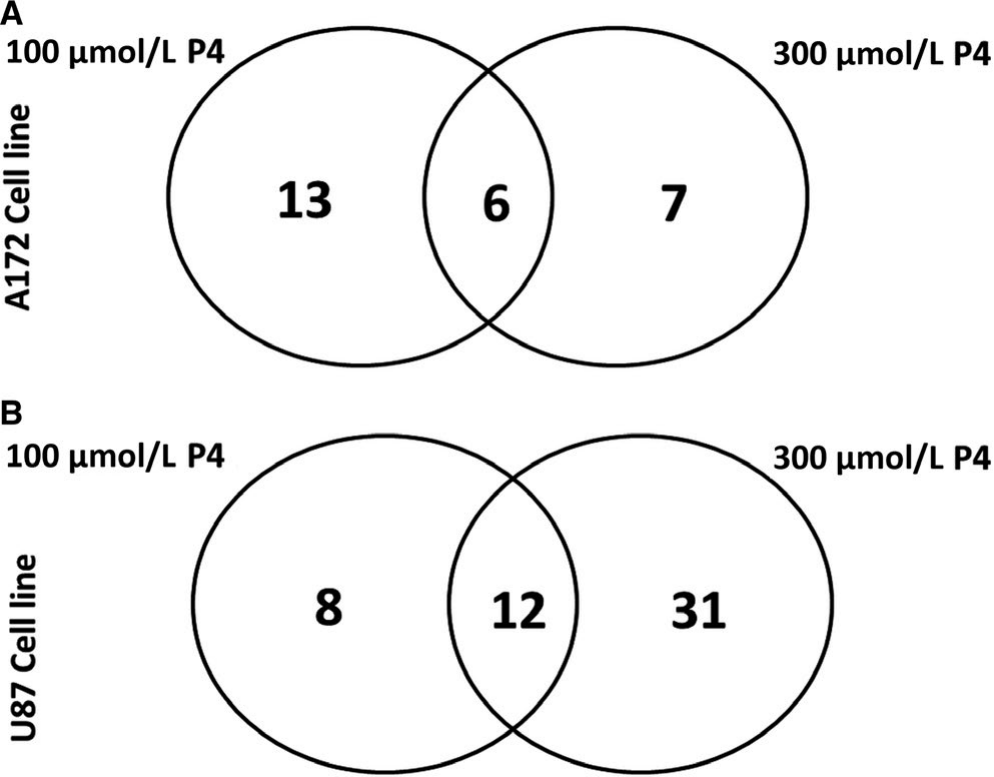
Venn diagram depicting differentially expressed proteins in (A) 172 cell line (B) U87 cell line was identified by mass spectrometry

Comparison of protein levels in two different GBM cell lines (U87 and A172) each treated with 100 µM P4, 300 µM P4, or without P4 was conducted, using a total of three gels per category. Each GBM cell line treated with P4 was compared with the same cell line that was not treated with P4. Representative gel images are shown in Figure 2. In total, we identified 26 differentially expressed (fc > 2) proteins in A172 cell lines treated with P4 (100 µM and 300 µM) versus A712 cell lines without P4 (Table 1 and Figure 3). A Venn diagram shows the common differentially expressed proteins in A172 cells treated with different P4 doses (100 µM P4 versus without P4 and 300 µM P4 versus without P4) (Figure 4). Of the 26 differentially expressed proteins in P4‐treated A172 cells, six proteins were found to be common in all comparisons; and only 13 and 7 proteins were unique in the A172 cell line treated with 100 µM and 300 µM P4, respectively (Table 1). The common proteins in P4‐treated A172 cells were alpha‐enolase, protein disulfide‐isomerase A6, heat shock 71 kDa cognate protein, mitochondrial ATP synthase subunit beta, glyceraldehyde‐3‐phosphate dehydrogenase, and mitochondrial ornithine aminotransferase. Of the six common proteins detected in A172 cells treated with either 100 µM or 300 µM P4, five proteins were upregulated; and only mitochondrial ornithine aminotransferase was downregulated (*P* < .05).

**TABLE 1:** Identifications of proteins that were significantly different between whole‐cell lysates of 100 µM P4 versus control and 300 µM P4 vs control in A172 cell line

Spot ID	UniProt ID	Gene	Protein name	Status
9401	P04075	ALDOA	Fructose‐bisphosphate aldolase A	Upregulated in 100 µM P4
9306	P21796	VDAC1	Voltage‐dependent anion‐selective channel protein 1	
1201	P61981	1433G	14‐3‐3 protein gamma	Downreguated in 100 µM P4
1201	P63104	1433Z	14‐3‐3 protein zeta/delta	
1505	P08670	VIME	Vimentin	
4901	P55072	TERA	Transitional endoplasmic reticulum ATPase	
8705	P04040	CATA	Catalase	
8705	P14618	KPYM	Pyruvate kinase PKM	
9501	P68104	EF1A1	Elongation factor 1‐alpha 1	
6709	P49368	TCPG	T‐complex protein 1 subunit gamma	
6802	P02545	LMNA	Prelamin‐A/C	
7406	P61163	ACTZ	Alpha‐centractin	
7406	P49411	EFTU	Elongation factor Tu, mitochondrial	
1403	P0DN37	PAL4G	Peptidyl‐prolyl cis‐trans isomerase A‐like 4G	Upregulated in 300 µM P4
1605	P26440	IVD	Isovaleryl‐CoA dehydrogenase	
3704	P10809	CH60	60 kDa heat shock protein, mitochondrial	
5107	P09211	GSTP1	Glutathione S‐transferase P	
5107	P28070	PSB4	Proteasome subunit beta type‐4	
3604	Q8NHM5	KDM2B	Lysine‐specific demethylase 2B	Downregulated in 300 µM P4
3803	P11021	BIP	Endoplasmic reticulum chaperone BiP	
2506	P06576	ATPB	ATP synthase subunit beta, mitochondrial	Upregulated in both 100 µM P4 and 300 µM P4
2506	Q15084	PDIA6	Protein disulfide‐isomerase A6	
8506	P06733	ENOA	Alpha‐enolase	
4808	P11142	HSP7C	Heat shock cognate 71 kDa protein	
6507	P04181	OAT	Ornithine aminotransferase, mitochondrial	Downregulated in both 100 µM P4 and 300 µM P4

**FIGURE 5:**
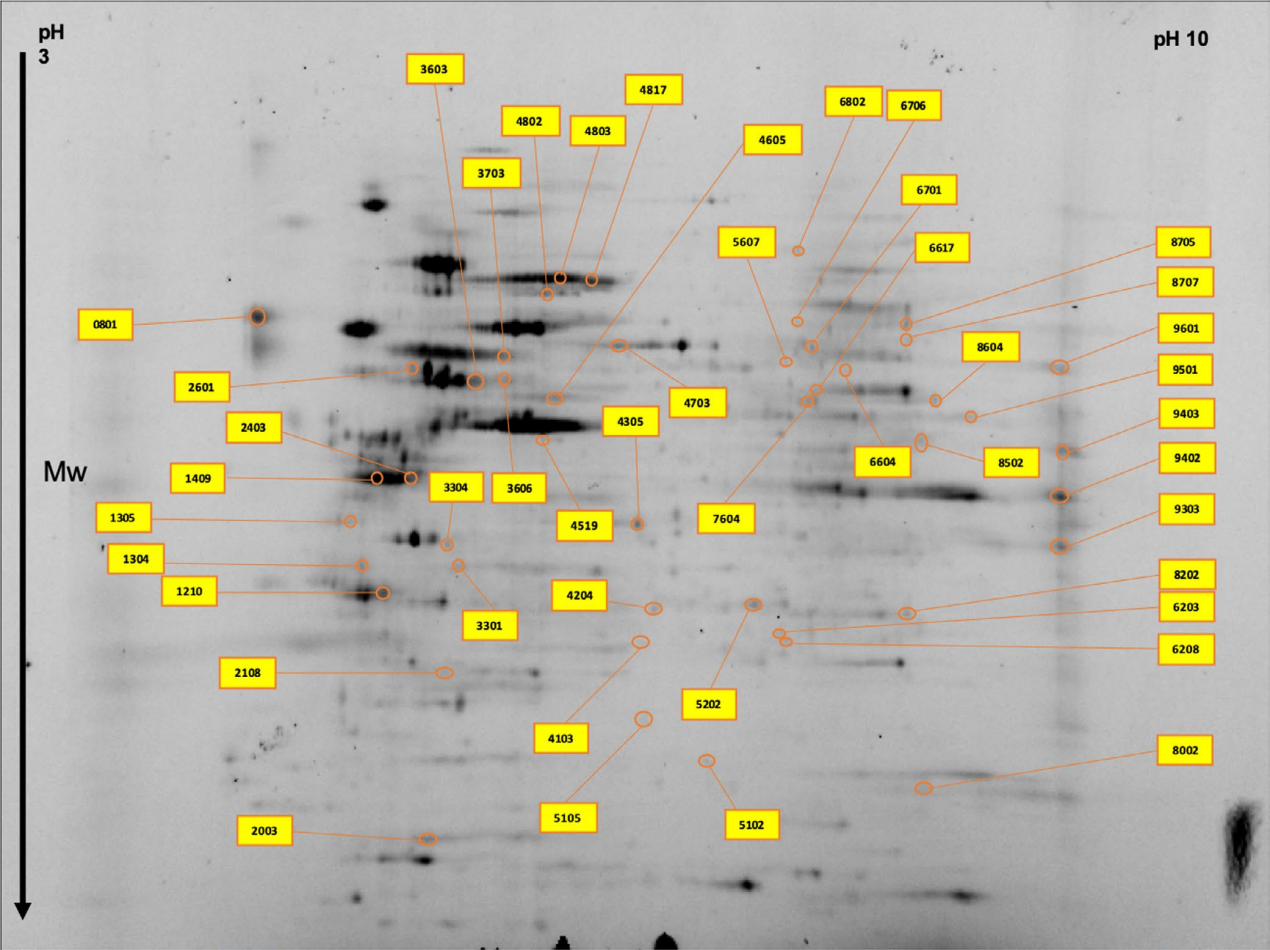
Significantly altered protein spots in the U87 cell line master gel image obtained by two‐dimensional gel electrophoresis proteomics analysis

In U87 cell lines treated with P4 (100 and 300 µM), we identified 51 differentially expressed (fc > 2) proteins when compared with the U87 cell line without P4 treatment (Table 2 and Figure 5). The Venn diagram in Figure 4 shows the common proteins (n = 12) detected in both U87 cell lines treated with 100 or 300 µM P4. We identified 8 and 31 unique proteins in U87 cell lines treated with 100 and 300 µM P4, respectively (Table 2). Of the 12 common proteins, 11 proteins were upregulated; only mitochondrial 60 kDa heat shock protein was downregulated (*P* < .05). Next, we compared differential protein expressions in A172 and U87 cell lines treated with either 100 or 300 µM P4. According to the Venn diagram (Figure 4), isovaleryl‐CoA dehydrogenase was upregulated in both cell lines when treated with 300 µM P4 and T‐complex protein 1 subunit gamma was downregulated in both cell lines when treated with 100 µM P4. Although no proteins were common in both cell lines when treated with 100 and 300 µM P4, three proteins were found to be common in a U87 cell line with 300 µM P4 and an A172 cell line with both 100 and 300 µM P4. These common proteins were identified as follows: alpha‐enolase, mitochondrial ATP‐synthase subunit beta, and heat shock cognate 71 kDa protein.

**TABLE 2:** Identifications of proteins that were significantly different between whole‐cell lysates of 100 µM P4 vs control and 300 µM P4 vs control in U87 cell line

Spot ID	UniProt ID	Gene	Protein name	Status
4605	P52597	HNRPF	Heterogeneous nuclear ribonucleoprotein F	Upregulated in 100 µM P4
5102	P15531	NDKA	Nucleoside diphosphate kinase A	
4519	Q9UJZ1	STML2	Stomatin‐like protein 2, mitochondrial	
3603	Q5JTZ9	SYAM	Alanine‐tRNA ligase, mitochondrial	Downregulated in 100 µM P4
7604	P26641	EF1G	Elongation factor 1‐gamma	
6802	P15311	EZRI	Ezrin	
7604	Q9UQ80	PA2G4	Proliferation‐associated protein 2G4	
6706	P49368	TCPG	T‐complex protein 1 subunit gamma	
8604	P06733	ENOA	Alpha‐enolase	Upregulated in 300 µM P4 Upregulated in 300 µM P4
4305	P12429	ANXA3	Annexin A3	
3304	P08758	ANXA5	Annexin A5	
9403	P00505	AATM	Aspartate aminotransferase, mitochondrial	
9601	P25705	ATPA	ATP synthase subunit alpha, mitochondrial	
2601	P06576	ATPB	ATP synthase subunit beta, mitochondrial	
3301	O00299	CLIC1	Chloride intracellular channel protein 1	
2108	Q13185	CBX3	Chromobox protein homolog 3	
9501	O75390	CISY	Citrate synthase, mitochondrial	
6203	P30040	ERP29	Endoplasmic reticulum resident protein 29	
8604	P07954	FUMH	Fumarate hydratase, mitochondrial	
9402	P04406	G3P	Glyceraldehyde‐3‐phosphate dehydrogenase	
8202	P62826	RAN	GTP‐binding nuclear protein Ran	
4803	P11142	HSP7C	Heat shock cognate 71 kDa protein	
2403	P07910	HNRPC	Heterogeneous nuclear ribonucleoprotein C1/C2	
5607	P31943	HNRH1	Heterogeneous nuclear ribonucleoprotein H	
8705	P14866	HNRPL	Heterogeneous nuclear ribonucleoprotein L	
9303	Q32P51	RA1L2	Heterogeneous nuclear ribonucleoprotein A1‐like 2	
8502	P26440	IVD	Isovaleryl‐CoA dehydrogenase, mitochondrial	
4802	Q03252	LMNB2	Lamin‐B2	
6701	Q10713	MPPA	Mitochondrial‐processing peptidase subunit alpha	
4202	O75489	NDUS3	NADH dehydrogenase	
1305	P12004	PCNA	Proliferating cell nuclear antigen	
6617	P50395	GDIB	Rab GDP dissociation inhibitor beta	
1210	Q96T23	RSF1	Remodeling and spacing factor 1	
6606	Q9Y265	RUVB1	RuvB‐like 1	
4802	Q9Y4G6	TLN2	Talin‐2	
3606	Q8NBS9	TXND5	Thioredoxin domain‐containing protein 5	
1304	P06753	TPM3	Tropomyosin alpha‐3 chain	
801	P27797	CALR	Calreticulin	Downregulated in 300 µM P4
4703	P30101	PDIA3	Protein disulfide‐isomerase A3	
8707	P47895	AL1A3	Aldehyde dehydrogenase family 1 member A3	Upregulated in both 100 µM P4 and 300 µM P4
5202	P30084	ECHM	Enoyl‐CoA hydratase, mitochondrial	
2003	P09382	LEG1	Galectin‐1	
4103	P09211	GSTP1	Glutathione S‐transferase P	
1409	P06748	NPM	Nucleophosmin	
8002	P62937	PPIA	Peptidyl‐prolyl cis‐trans isomerase A	
6208	P30041	PRDX6	Peroxiredoxin‐6	
4817	P38646	GRP75	Stress‐70 protein, mitochondrial	
5105	P30048	PRDX3	Thioredoxin‐dependent peroxide reductase, mitochondrial	
3703	P10809	CH60	60 kDa heat shock protein, mitochondrial	Downregulated in both 100 µM P4 and 300 µM P4

### 3.3 Biological functions and associated pathways of differentially regulated proteins

We analyzed the biological functions and corresponding pathways affected by P4 administration on U87 and A172 GBM cell lines. A total of 70 differentially expressed proteins identified from 2D gel electrophoresis were functionally assessed using pathfindR. In pathfindR, the pathways associated with the differentially expressed proteins were analyzed using the corresponding genes of those proteins. Therefore, we matched 9 and 32 unique genes in the U87 cell line with 100 µM P4 and 300 µM P4, respectively, with the differentially expressed proteins. Also, 11 genes were found to be common between 100 µM P4 and 300 µM P4 administered the U87 cell line. Moreover, we matched 14 and 8 unique genes in the A172 cell line with 100 µM P4 and 300 µM P4, respectively, with the differentially expressed proteins. Five genes were found to be common between 100 µM P4 and 300 µM P4 administered A172 cell line.

We compared each cell line with one another when treated with 100 and 300 µM P4. High‐dose progesterone treatments induced a plethora of pathways both in U87 and A172 human GBM cells. Top Reactome pathways associated with differentially expressed proteins in different GBM cells with 100 and 300 µM P4 administrations are given in Tables 3, 4, 5, 6.

**TABLE 3:** Top Reactome pathways in U87 cell line with 100 µM P4 administration

	Upregulated	Downregulated
Detoxification of Reactive Oxygen Species	GSTP1, PRDX3, PRDX6	
Gene and protein expression by JAK‐STAT signaling after Interleukin‐12 stimulation	HNRNPF, HSPA9, PPIA	
TFAP2A acts as a transcriptional repressor during retinoic acid induced cell differentiation	NPM1	HSPD1

**TABLE 4:** Top Reactome pathways in U87 cell line with 300 µM P4 administration

Pathway	Upregulated	Downregulated
Antigen Presentation: Folding, assembly and peptide loading of class I MHC	HSPA5	CALR, PDIA3
ATF6 (ATF6‐alpha) activates chaperone genes	HSPA5	CALR
Cellular responses to stress	GSTP1, PRDX3, PRDX6, HSPA5, HSPA8, HSPA9	
Detoxification of Reactive Oxygen Species (*subpathway of Cellular responses to stress*)	GSTP1, PRDX3, PRDX6	
Cellular response to heat stress (*subpathway of Cellular responses to stress*)	HSPA5, HSPA8, HSPA9	
Chromosome Maintenance	NPM1, PCNA, RSF1, RUVBL1	
Nucleosome assembly (*subpathway of Chromosome Maintenance*)	NPM1, RSF1, RUVBL1	
Citric acid cycle (TCA cycle)	CS, FH	
Glucose metabolism	ENO1, GAPDH, GOT2	
Mitochondrial protein import	CS, ATP5F1B, HSPA9, ATP5F1A, PMPCA	HSPD1
mRNA Splicing—Major Pathway	HNRNPC, HNRNPH1, HNRNPL, HSPA8	
TFAP2A acts as a transcriptional repressor during retinoic acid induced cell differentiation	NPM1	HSPD1
TP53 Regulates Transcription of Cell Cycle Genes	NPM1, PCNA	
Unfolded Protein Response (UPR)	HSPA5	CALR

**TABLE 5:** Top Reactome pathways in A172 cell line with 100 µM P4 administration

Pathway	Upregulated	Downregulated
Apoptosis		LMNA, VIM, YWHAG, YWHAZ
Cellular responses to stress	HSPA8	ACTR1A, CAT, EEF1A1, VCP
Cellular response to heat stress (*subpathway of Cellular responses to stress*)	HSPA8	EEF1A1, VCP
Chk1/Chk2(Cds1)‐mediated inactivation of Cyclin B:Cdk1 complex		YWHAG, YWHAZ
Glucose metabolism	ALDOA, ENO1, GAPDH	PKM
Protein methylation	HSPA8	EEF1A1, VCP
Regulation of mRNA stability by proteins that bind AU‐rich elements	HSPA8	YWHAZ
TP53 Regulates Metabolic Genes		YWHAG, YWHAZ

**TABLE 6:** Top Reactome pathways in A172 cell line with 300 µM P4 administration

Pathway	Upregulated	Downregulated
AUF1 (hnRNP D0) binds and destabilizes mRNA	HSPA8, PSMB4	
Cellular responses to stress	GSTP1, HSPA8, PSMB4	HSPA5
Glucose metabolism	ENO1	GAPDHS
Protein methylation	HSPA8	

## 4 DISCUSSION

P4 is a cycling ovarian steroid hormone that promotes glandular differentiation of the endometrial epithelium to prepare the uterus for a possible pregnancy.^2^ Even though pregnancy may accelerate GBM growth, parity lowers the lifelong risk of GBM, which may associate with the fact that high dose and prolonged exposure to P4 may eliminate cells leading to GBM growth.^2^ Based on fine structural features, P4 is proposed to induce an oligodendroglial differentiation shift in GBM cells.^7^ Despite the P4 receptor is a steroid receptor that has the highest correlation with glial tumor grade ^2^, ^15^; there exist surprisingly few studies which assessed the effects of P4 on the biological behavior of GBMs.

P4 is a neurosteroid hormone synthesized within the brain tissue, yet its levels in malignant glial tumors are lesser (almost 28‐fold) than benign brain tissues. This suggests that it might be an “unwanted” mediator in GBMs.^2^, ^6^ Despite this fact, studies were conducted to see whether the blockage of P4 synthesis with PR‐antagonist mifepristone was capable to reduce the growth of GBMs since pregnancy propagates GBM growth similar to meningiomas.^6^ Indeed, there was some activity of mifepristone to reduce growth stimulation mediated by low dose (10 nM) of P4 albeit at concentrations 1000 times higher than P4.^6^, ^16^, ^17^, ^18^ However, there were some problems in proposing mifepristone as a candidate for GBM treatment. For example, it is not available in every country due to ethical concerns since it might be employed to abort a pregnancy in the late term. Moreover, it might lead to prolonged uterine hemorrhage, adrenal insufficiency, nausea, and pelvic infections.^6^ Therefore, it is proposed that another PR‐antagonist used for emergency contraception, ulipristal may be a safe option ^6^; and indeed, we witnessed some activity of ulipristal in reducing GBM growth (our unpublished observations). Nonetheless, applying P4 at high doses seems physiological, as P4 levels during pregnancy increase about 200‐fold, and treatment with very large doses of P4 is tolerable and still the preferred choice in the treatment of early endometrial cancer, in patients who wish to preserve their fertility.^6^ Additionally, there is a growing interest in the potential antineoplastic of P4 in GBM.^5^, ^8^, ^9^ Recently, it has been shown that P4 is more effective than conventional steroids to alleviate brain tumor edema and prolongs survival in rats with orthotopic GBM allografts.^9^ This is a very meaningful finding considering that conventional steroids increase blood glucose, may propagate GBM growth, and even reduce survival in a clinical setting.^19^

One recent study proposed that P4‐induced growth suppression in GBM cells mainly occurred via suppression of glycolytic pathways and inducing senescence.^8^ As will be outlined below, some of our findings are parallel to these concepts. We selected to study two different GBM cell lines (U87 and A172) with different genetic constitutions to elucidate P4 actions in a more detailed manner. For example, the U87 cell line harbors a wild‐type p53 gene, the A172 cell line is p53 mutant.^20^ The U87 GBM cells invade de‐epithelialized tracheas with increased matrix metalloprotease‐3 (MMP‐3) activity, but A172 cells are incapable to do so.^20^

### 4.1 Changes regarding senescence proteins in glioma

In both A172 and U87 GBM cells, 100 µM P4 reduced T‐complex protein 1 subunit gamma (TCPG, UniProt ID: P49368) levels. TCPG belongs to the chaperonin‐containing T‐complex (TRiC) which regulates the folding of WRAP53/TCAB1 and provides telomere maintenance.^21^ The proliferation ability of a cell can be determined by the telomeres. The shortage of telomeres in cell division affects the Hayflick limit (regeneration limit) of cells. This also influences the entrance to the senescence.^22^ Tumor cells confront this problem by protecting telomeres using a mechanism driven by a telomerase complex. For high‐grade gliomas with a potential of unlimited proliferation, it is also fundamental to maintain the telomere lengths.^23^ Therefore, the reduction of a protein involved in the maintenance of telomeres may associate with the P4‐induction of senescence in gliomas. In A172 GBM cells, 100 µM P4 reduced the levels of *LMNA*/LMNA (Prelamin A/C), which has a role in the proper structuring of nuclear membrane lamellae. A‐type lamins maintain the telomere length, and their loss results in telomere shortening.^23^ In colorectal carcinoma, high expression of A‐type lamins enhances the mortality risk.^24^

Decreased levels of YWHAZ protein may provide further evidence for a senescence induction by P4 in GBM cells. In A712 GBM cells, 100 µM P4 reduced levels of *1433Z*/YWHAZ (14‐3‐3 Protein Zeta), which involves in regulation of apoptosis; Chk1/Chk2(Cds1) mediated inactivation of Cyclin B:Cdk1 complex; and TP53 regulation of metabolic genes. In general, 14‐3‐3 proteins suppress apoptosis ^24^ and act as potential oncogenes.^25^ YWHAZ also involved in controlling the mRNA stability by AU‐rich element‐binding proteins. Decreased levels of YWHAZ may be beneficial, as its depletion induces senescence in GBM cells.^26^ YWHAZ also promotes ovarian cancer metastasis via vimentin‐dependent pathways and the addition of P4 decreased vimentin levels in our experiments.^27^ In A172 GBM cells, 300 µM P4 reduced vimentin levels (*VIME*/VIM UniProt ID: P08670). *VIME*/VIM is a type III intermediate filament (IF) protein present mostly in mesenchymal cells, which regulates the anchoring and position of the cytosolic organelles. Vimentin attaches to the nucleus, ER, and mitochondria and is expressed in malignant cells during the epithelial‐mesenchymal transition (which increases tumor aggressiveness).^28^ In cancer cells, enhancement of vimentin solubility with cholesterol‐lowering statin drugs reduces malignant characteristics which associate with enhanced apoptosis.^28^ Reduction of *VIME*/VIM levels by P4 represents a beneficial effect, as high *VIME*/VIM expression correlates with a high grade in gliomas while low vimentin levels associated with better survival and temozolomide response in GBM.^29^

### 4.2 Changes regarding proteins involving in cellular responses to stress

In our study, *CH60*/HSPD1/HSP60 (60 kDa heat shock protein, UniProt ID: P10809) chaperone was decreased in 100 and 300 µM P4‐exposed U87 GBM cells. *CH60*/HSPD1/HSP60 is a chaperon regulating the import of proteins into mitochondria and assembly of macromolecules.^30^ siRNA mediated HSP60 silencing, induces CypD‐associated transition of mitochondrial permeability, caspase‐associated apoptosis, and prevention of in vitro and intracranial GBM growth.^31^ In our analyses, *CH60*/HSPD1/HSP60 also emerged as a component of the pathway enrichment analysis regarding TFAP2A mediates retinoic acid‐induced cellular differentiation via acting as a transcriptional repressor. Interestingly, *CH60*/HSPD1/HSP60 involves in retinoic acid resistance in acute promyelocytic leukemia cells.^31^ Hence, the reduction of *CH60*/HSPD1/HSP60 may increase differentiation‐inducing efficacy of retinoids. Indeed, P4 also decreased the levels of calreticulin, which inhibits retinoic acid‐induced cell differentiation.^32^ As retinoic acid signaling enhances differentiation and temozolomide sensitivity of GBM cells, a simultaneous decrease in *CH60*/HSPD1/HSP60 and calreticulin may enhance differentiation and growth suppression in GBM.^29^, ^33^ Administration of 300 µM P4 increased the levels of heat shock 71 kDa Cognate Protein (*HSP7C*/HSPA8) both in U87 and A172 GBM cells. *HSP7C*/HSPA8 involves versatile pathways including cellular responses to stress, protein methylation, mRNA splicing, and regulation of mRNA stability.^34^, ^35^ We presume that this increase reflects a self‐defense mechanism of cells against metabolic stress, as *HSP7C* is a molecular chaperone protein which expression is triggered in squamous cell carcinomas when they become resistant to cisplatin.^36^

### 4.3 Proteins of energy metabolism influenced by P4

Ornithine‐delta‐aminotransferase (OAT) is a nucleus‐encoded, mitochondrial enzyme that converts ornithine to glutamate semialdehyde.^37^ When compared with the benign hepatic tissue, the OAT level of Morris hepatoma was found to be approximately 15 times higher.^38^ OAT stimulated the proliferation, migration, and invasion and blocked the apoptosis, while the lack of OAT reduced the growth and metastasis of lung cancer xenograft.^39^ Hence, P4 reduction of OAT levels may be beneficial in terms of growth and invasion suppression in GBM. Three hundred µM P4 increased the levels of the mitochondrial ATP synthase subunit beta (ATP5F1B, UniProt ID: P06576). Mitochondrial membrane ATP synthase synthesizes ATP from ADP in the existence of a proton gradient in the cell membrane. The downregulation of the ATP5F1B is a hallmark of most human cancers and the subsequent decrease in mitochondrial bioenergetics causes a glucose demand in malignancies.^40^

Warburg effect defines the preference of cancer cells to employ anaerobic glycolysis for producing energy even an adequate level of oxygen is present. Hence, P4 might counteract the Warburg effect in tumors and the associated aggressiveness via increasing the ATP5F1B. Indeed, low levels of ATP5F1B associated with worse prognosis in colorectal cancer treated with 5‐Fluorouracil ^41^ and resistance to adriamycin in leukemia cells.^42^ The anticancer hormone melatonin upregulated ATP5F1B expression in ovarian cancer.^43^ Interestingly, ATP5F1b also exists in cell membranes of cancer cells, and potential antibodies targeted to ATP5F1b resulted in better survival in mesothelioma.^44^ Hence, the enhancement of this protein may also increase the antigenicity of GBM cells.

Hundred µM P4 decreased Pyruvate Kinase M (*PKM*/PKM, UniProt ID: P14618) levels in A172 GBM cells. *PKM*/PKM is an enzyme that functions in the glycolytic pathway and catalyzes the pyruvate formation from phosphoenolpyruvate (PEP) by producing ATP. M1 isoform is mainly found in muscle, heart, and brain, while M2 exists in early fetal tissues and most cancer cells. Both isoforms are synthesized from the same gene via differential splicing.^45^ Grade I‐III gliomas showed relatively high levels of PKM2 in RNA and protein levels, whereas GBMs were found to exert an almost 3‐ to 5‐fold increase in PKM2 levels.^46^ As high glycolysis is necessary for tumor growth and survival, a decrease in *PKM*/PKM with P4 may be an important mechanism of growth suppression. In our analysis, YWHAG and YWHAZ also emerged as components of proteomic pathways providing membrane translocation of GLUT4, which mediates glucose transport of GBM cells.^47^ Hence, P4 at high dosages may block glucose transport in GBM cells.

### 4.4 Progesterone and immune pathways

In A172 cells, 300 µM P4 decreased the levels of Protein Disulfide Isomerase A3 (PDIA3), which is involved in MHC Class‐I antigen processing. Overexpression of PDIA3 in diffuse glioma patients shows decreased survival. In vitro knockdown of PDIA3, decreased cell proliferation, triggered apoptosis, and lowered invasion of glioma cells.^48^ Hence, P4 reduction of PDIA3 may be both directly and indirectly (via immunological stimulation) beneficial in terms of GBM management when applied in vivo. In U87 GBM cells, 100 µM and 300 µM P4 increased the levels of peptidyl‐prolyl cis‐trans isomerase A (*PPIA*/PPIA, or: Cyclophilin A; UniProt ID: P62937). Peptidyl‐prolyl cis‐trans isomerase enzymes (PPIases) regulate cis‐trans isomerization in peptide bonds and thus, controls the folding of proteins. The expression of *PPIA*/PPIA is increased in the anterior cingulate cortex in the brain and its expression is associated with neuronal differentiation in marrow mesenchymal stem cells.^49^ In our analyses, PPIA was also revealed to involve in IL‐12 signaling. P4 stimulates IL‐12 synthesis within the ectocervical epithelia.^50^ P4 exerts both immunosuppressive and immunostimulatory effects in pregnancy, which might have implications for tumor immunology ^2^; and it remains to be elucidated whether P4 involves in the synthesis and downstream signaling of IL‐12 in GBM. It was previously shown that high‐dose MPA treatment caused a prominent lymphomonocytic infiltration around the C6 GBM tumor growing intracranially ^1^; and high‐dose P4 increased the antigenicity of GBM, which may be harnessed in future endocrine‐immune protocols for GBM treatment.

## 5 CONCLUSIONS

P4 hormone and its analogs are highly safe at very large doses and act anti‐neoplastic via different mechanisms than cytotoxic chemotherapeutics.^1^, ^2^ Therefore, hormone‐sensitizing agents can be developed to enhance the antineoplastic of P4 even further. Fortunately, there exist many preclinical and even clinical data that showed agents capable to enhance the antineoplastic of P4 analogs including MPA. Among these are the neuroleptic agent thioridazine,^51^ the antidiabetic agent metformin,^52^ and the cholesterol‐lowering agent bezafibrate,^53^ which are already being safely used for indications other than cancer. These agents can be easily reemployed for P4‐sensitization in GBM as their pharmacokinetics, therapeutic window, and toxicity levels are well established. Proteomic studies about P4’s anti‐neoplastic effects in GBM cells would provide invaluable insights about a putative endocrine treatment approach and pave the development of further combinations including P4‐analogs and P4‐sensitizers.

## CONFLICT OF INTEREST

The authors declare that the research was conducted in the absence of any commercial or financial relationships that could be construed as a potential conflict of interest.

## AUTHORS’ CONTRIBUTIONS

AO and İE were involved in all aspects of design, interpretation, and writing. MAA and YU led laboratory analysis and wrote the manuscript. MCY contributed to the proteomic analyses. İK involved in the cell culture experiments. OO and US contributed to the bioinformatic analyses. All authors provided critical feedback and helped shape the research, analysis, and manuscript.

## Data Availability

The data that support the findings of this study are available from the corresponding author upon reasonable request.
